# Disentangling potential genotypes for macro and micro nutrients and polymorphic markers in Chickpea

**DOI:** 10.1038/s41598-023-37602-2

**Published:** 2023-07-03

**Authors:** Neha Mittal, Juhi Bhardwaj, Shruti Verma, Rajesh Kumar Singh, Renu Yadav, D. Kaur, Akshay Talukdar, Neelam Yadav, Rajendra Kumar

**Affiliations:** 1 Department of Biotechnology, Meerut Institute of Engineering & Technology, Meerut, 250005 India; 2 NCoE-SAM, Department of Pediatrics, KSCH, Lady Hardinge Medical College, New Delhi, 110001 India; 3 Division of Genetics, ICAR-Indian Agricultural Research Institute, New Delhi, 110012 India; 4 AIOA, Amity University, Noida, UP 201313 India; 5 Centre for Food Technology, University of Allahabad, Prayagraj, UP 211002 India

**Keywords:** Biochemistry, Genetics, Plant sciences

## Abstract

The present investigation was conducted to assess the nutritional diverseness and identify novel genetic resources to be utilized in chickpea breeding for macro and micro nutrients. The plants were grown in randomized block design. Nutritional and phytochemical properties of nine chickpea genotypes were estimated. The EST sequences from NCBI database were downloaded in FASTA format, clustered into contigs using CAP3, mined for novel SSRs using TROLL analysis and primer pairs were designed using Primer 3 software. Jaccard’s similarity coefficients were used to compare the nutritional and molecular indexes followed by dendrograms construction employing UPGMA approach. The genotypes PUSA-1103, K-850, PUSA-1108, PUSA-1053 and the EST-SSR markers including the 5 newly designed namely ICCeM0012, ICCeM0049, ICCeM0067, ICCeM0070, ICCeM0078, SVP55, SVP95, SVP96, SVP146, and SVP217 were found as potential donor/marker resources for the macro–micro nutrients. The genotypes differed (*p* < 0.05) for nutritional properties. Amongst newly designed primers, 6 were found polymorphic with median PIC (0.46). The alleles per primer ranged 1 to 8. Cluster analysis based on nutritional and molecular diversities partially matched to each other in principle. The identified novel genetic resources may be used to widen the germplasm base, prepare maintainable catalogue and identify systematic blueprints for future chickpea breeding strategies targeting macro–micro nutrients.

## Introduction

Chickpea, is a self-pollinating diploid (2n = 2x = 16) with genome size 1C = 740 Mbp^1^. It consists of remarkable attributes like wide climate adaptation, low production cost and having an ability to be applied in crop alternation and atmospheric nitrogen fixation. Chickpea is a noteworthy legume plant for sustainability of agriculture system^2^. Despite having little productivity especially due to Fe deficiency induced by lime, Chickpea, is cultivated on large areas of world^2^. It is the second most significant pulse (after dry beans) crop which is grown mainly in the arid and semi-arid regions, grown over 40 countries representing all the continents, with 13.72 million hectares (MHa) total harvested area, 1038.4 kg per hectare (Kg/Ha) total yield and 14.25 million tonnes (MT) total production^3^. Developing countries hold largest share (95%) in terms of area, production and consumption of chickpeas. During the span of last 30 years (1989–2019), worldwide chickpea area amplified by 138.56%, yield by 143.29% and production by 198.53%^3^. Presently, it is cultivated in several countries with the largest harvested area of 9.55 MHa by India followed by Pakistan, Russian Federation, Turkey, Myanmar etc^3^. Currently, India represents as the principal chickpea producer contributing around 69.76% of the global production followed by Turkey, Russian Federation, Myanmar and Pakistan considered as the top five major world producers^3^. The main pulse crops i.e., beans, peas, and chickpeas account for around 64.17% of global pulse production with chickpea accounting for nearly 16.12%^3^. In India during 2018–19, it was cultivated in 9.44 MHa area with 10.13 MT total production and 1073 kg/Ha yield. Madhya Pradesh ranked 1st with highest acreage of 3.43 MHa followed by Rajasthan, Maharashtra, Karnataka and Uttar Pradesh. The highest production of 4.61 MT was contributed by Madhya Pradesh followed by Rajasthan, Maharashtra and Uttar Pradesh. The highest yield of 1344 kg/Ha was produced by Madhya Pradesh followed by Gujarat (1324), Uttar Pradesh (1272) and Rajasthan (1103)^4^. However, as per very recently released 3rd advance estimates, India expects 12.63 MT of total chickpea production during 2020–21^5^.

Owing to different quality and quantity traits, chickpea owns huge variations which assist breeders to develop advanced lines and release better-quality varieties^6^. Chickpea is one of the earliest cultivated edible grain legumes^7^. It’s about 7500-year-old remnants usually found in the Middle East^7^. The nutrients like fat, protein and carbohydrate which are required in larger quantities and empower us with energy are known as macronutrients. However, the nutrients like vitamins and minerals which are required in very small quantities but equally important for maintenance of our health. We usually acquire our micronutrients and macronutrients together. Chickpea serves as an ideal crop for human consumption owing to its high nutritive values for protein (17–24%), carbohydrates (41–50.8%), vitamins (Vitamin C, Thiamin, Riboflavin, Niacin, B6, Folate, Vitamin B12, Vitamin A, D, E and K), minerals (Calcium,Iron, Magnesium, Phosphorous, Potassium, sodium, Zinc, Copper, Manganese and selenium) and unsaturated acids like linoleic, oleic etc^8^. It is important to note that identifying potential genotypes is very much vital, when huge accessions of crop germplasm are being considered. Hence, newly evolved cultivar is to be registered and purity of the variety has to be ascertained. DNA markers offer very efficient and well-grounded techniques for assessing the genomic changeability and affiliations among germplasm lines. Hence, DNA markers are considered very effective tools for assessing genomic variations and learning developmental association ships^9^. In plant genomes, PCR based techniques and microsatellite sequences facilitate to analyze the genomic diversity. Genomic analysis procedures using DNA polymorphism have been progressively used to describe and classify a novel germplasm for use in the crop breeding process^10^. Environmental factors and growth practices affect the morphological and nutritional markers, whereas DNA markers remain unaffected by environmental conditions.

The present study utilized expressed sequence tags (ESTs) derived simple sequence repeats (SSRs) or also known as expressed sequence tag—simple sequence repeat (EST-SSR) markers to assess the pattern and the presence of genomic changeability and congruence among the genotypes. Thus, findings would be helpful in identifying and differentiating numerous genotypes for local consumption or for exportation purpose, selection of diverse parents and devise competent approaches for the efficient management of the genetic resources and to widen the germplasm base which could be used in the forthcoming nutrition rich chickpea breeding plans.

## Results

### Macro and micro nutrients’ proximate composition analysis

We estimated average values for nutritional compositions encompassing twelve macro and micro nutrient parameters viz; ash (%), moisture (%), protein (g/100 g), fat (g/100 g), carbohydrate (g/100 g), fibre (g/100 g), TPC (mg/100 g), phytate (mg/100 g), antiradical activity (%), tannin (%), iron (mg/100 g) and zinc (mg/100 g). The proximate compositions (Table 1) varied highly from one genotype to another. The ash content was found to be maximum (3.9%) for PUSA-362 and minimum (3.0%) for PUSA 1053. Similarly, protein was found to be significantly higher (31 g/100 g) for PUSA 1108 and lower (18 g/100 g) for PUSA 1103, whereas carbohydrate was maximum (68.10 g) for PUSA 1103 and minimum (54.4 g) for PUSA 1088 and PUSA 1105. The fibre content was found to be highest (5.8 mg/100 g) for K 850 and lowest (3.4 mg/100 g) for PUSA 1108. The maximum (255 mg/100 g) TPC was found in K850, whereas minimum (101 mg/100 g) was found in PUSA 1053. The tannins were found to be highest (0.22%) for JG 62 and minimum (0.07%) for PUSA 1053. Similarly, genotype JG74 showed highest phytate content (1100 mg/100 g).

**Table 1:** Macro–micro nutritional analysis for 12 traits in 9 chickpea varieties.

Nutrients → Varieties↓	Ash (%)	Moisture (%)	Protein (g/100 g)	Fat (g/100 g)	Carbohy-drate (g/100 g)	Fibre (g/100 g)	TPC (mg/100 g)	Phytate (mg/100 g)	Anti-radical activity	Tannin (%)	Iron (mg/100 g)	Zinc (mg/100 g)
PUSA-362	3.9 ± 0.36	11.2 ± 0.03	22 ± 1.52	4.1 ± 0.28	60.7 ± 0.07	5.7 ± 0.20	212 ± 0.57	800 ± 0.66	85 ± 2.64	0.20 ± 0.00	5.8 ± 0.17	2.8 ± 0.20
K-850	3.2 ± 0.15	4.3 ± 0.12	23 ± 0.57	5.6 ± 0.10	58.6 ± 1.02	5.8 ± 0.26	255 ± 1.60	804 ± 1.15	88 ± 0.76	0.21 ± 0.01	8.6 ± 0.20	5.3 ± 0.32
PUSA-1105	3.5 ± 0.46	9.3 ± 0.32	28 ± 1.00	6.8 ± 0.35	54.5 ± 0.29	3.8 ± 0.10	138 ± 1.00	605 ± 0.58	68 ± 1.00	0.08 ± 0.02	10.3 ± 0.20	5.5 ± 0.20
PUSA-1108	3.6 ± 0.28	15 ± 0.55	31 ± 0.57	3.1 ± 0.25	55.3 ± 1.45	3.4 ± 0.17	178 ± 1.00	750 ± 0.58	84 ± 2.64	0.13 ± 0.02	6.1 ± 0.20	4.5 ± 0.20
PUSA-1103	3.2 ± 0.43	9.6 ± 0.23	18 ± 1.52	2.6 ± 0.28	68.1 ± 1.16	4.4 ± 0.45	203 ± 3.00	767 ± 0.58	85 ± 1.52	0.19 ± 0.01	7.4 ± 0.20	5.8 ± 0.10
JG-62	3.7 ± 0.30	10 ± 0.03	22 ± 1.52	5.3 ± 0.70	61.9 ± 0.03	3.5 ± 0.45	245 ± 1.52	804 ± 1.15	87 ± 3.60	0.22 ± 0.01	5.1 ± 0.26	2.7 ± 0.26
PUSA-1053	3.0 ± 0.11	11.0 ± 1.08	28 ± 1.00	5.0 ± 0.40	56.6 ± 0.48	3.7 ± 0.02	101 ± 1.60	598 ± 0.58	67 ± 3.05	0.07 ± 0.01	10.5 ± 0.45	6.2 ± 0.26
JG-74	3.3 ± 0.26	10.6 ± 0.22	22 ± 1.44	4.9 ± 0.05	61.2 ± 0.23	4.9 ± 0.10	223 ± 1.52	1100 ± 0.66	86 ± 1.00	0.18 ± 0.01	4.6 ± 0.20	3.6 ± 0.32
PUSA-1088	3.7 ± 0.05	10.2 ± 0.56	29 ± 0.57	5.2 ± 0.15	54.4 ± 0.83	4.1 ± 0.03	173 ± 1.52	669 ± 0.58	77 ± 3.60	0.12 ± 0.01	7.1 ± 0.22	2.2 ± 0.26

All data are expressed on a dry weight basis and represent the ± SE means of three replicates.

In vitro Antiradical activity was assessed as radical scavenging activity (%) of antioxidants (viz*.* TPC, tannins, flavonoids etc.). Variability among 9 genotypes was observed against relatively stable violet/purple colored DPPH oxidant (2, 2-diphenyl-1-picrylhydrazyl radical) (Table 1). The genotype K 850 possessed maximum (88%) antiradical activity, whereas minimum antiradical activity was obtained in the genotype PUSA 1053 (67%). Regarding minerals, the concentration of iron and zinc differed greatly among chickpea genotypes. As shown in Table 1, the iron concentration ranged between 4.6 to 10.5 mg/100 g, whereas zinc ranged between 2.2 and 6.2 mg/100 g. The genotypes PUSA1053 (10.5 mg/100 g), PUSA1105 (10.3 mg/100 g) and K 850 (8.6 mg/100 g) showed markedly high Fe levels in seeds, whereas the genotypes PUSA 362 (5.8 mg/100 g) and JG 74 (4.6 mg/100 g) showed lower levels. Similarly, zinc content was found higher in genotypes PUSA 1053 (6.2 mg/100 g) and PUSA 1103 (5.8 mg/100 g), whereas the genotype PUSA 1088 (2.2 mg/100 g) showed lowest concentration.

### Macro and micro nutrients’ based similarity versus dissimilarity analysis

Nutritional profile for macro and micro nutrient-based similarity analysis (Table 2) reflected negligible similarities coefficients (0.090) between PUSA-362 versus PUSA-1103, PUSA-362 versus JG-62, PUSA-362 versus JG-74, K-850 versus PUSA-1103, K-850 versus JG-62, PUSA-1105 versus PUSA-1053, JG-62 versus JG-74 and JG-62 versus PUSA-1088 respectively and no similarities were observed for other genotype pairs.

**Table 2:** Similarity matrix based on Jaccard’s coefficient values to show molecular and nutritional collinearities amongst the 9 chickpea varieties.

Nutritional → Molecular↓	PUSA-362	K-850	PUSA-1105	PUSA-1108	PUSA-1103	JG-62	PUSA-1053	JG-74	PUSA-1088
PUSA-362	1	0.00	0.00	0.00	0.09	0.09	0.00	0.09	0.00
K-850	0.91	1	0.00	0.00	0.09	0.09	0.00	0.00	0.00
PUSA-1105	0.94	0.87	1	0.00	0.00	0.00	0.09	0.00	0.00
PUSA-1108	0.89	0.86	0.92	1	0.00	0.00	0.00	0.00	0.00
PUSA-1103	0.94	0.87	0.93	0.92	1	0.00	0.00	0.00	0.00
JG-62	0.89	0.84	0.90	0.91	0.90	1	0.00	0.09	0.09
PUSA-1053	0.87	0.85	0.86	0.83	0.91	0.90	1	0.00	0.00
JG-74	0.90	0.85	0.87	0.90	0.93	0.94	0.93	1	0.00
PUSA-1088	0.93	0.87	0.94	0.91	0.94	0.87	0.90	0.92	1

**Figure 1:**
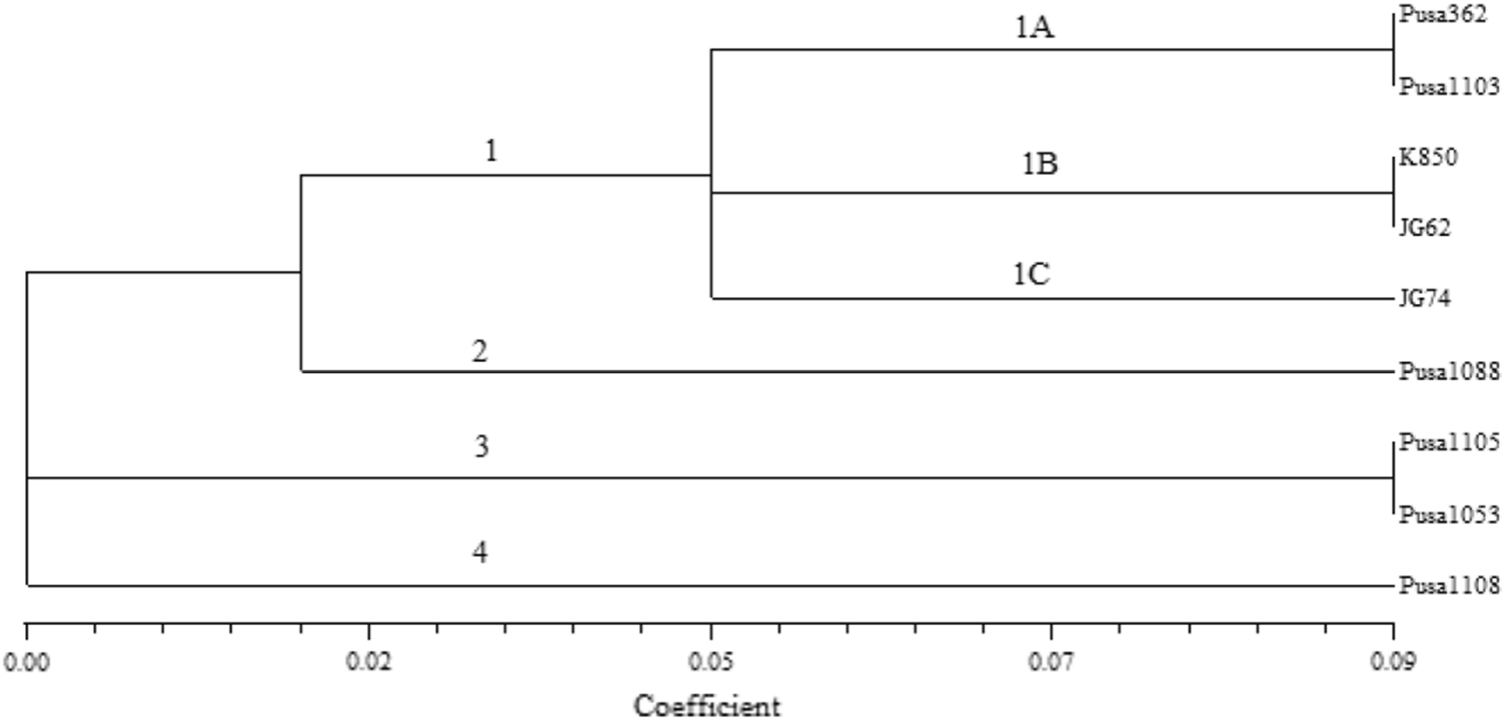
Macro–micro nutrients based dendrogram of 9 chickpea varieties constructed by UPGMA cluster analysis based on nutritional similarity indexes.

The similarity coefficients were applied to congregate the data as per UPGMA algorithm. The consequent phenogram clumped 9 genotypes towards four distinct conglomerations escorted by different sub clusters (Fig. 1). The Cluster-1 comprises 5 cultivars and those are further aligned into three sub clusters viz*.* 1A, 1B and 1C. The sub cluster 1A is represented by two genotypes PUSA-362 and PUSA-1103. The sub cluster 1B is represented by two genotypes K-850 and JG-62. The sub cluster 1C is represented by a single genotype JG-74. The cluster 2 is represented by a single genotype PUSA-1088. The cluster 3 is represented by two genotypes PUSA-1105 and PUSA-1053. The cluster 4 is represented by a single genotype PUSA-1108 and remains isolated at the end of the dendrogram.

### Molecular analysis

Genetic markers are extensively harnessed to discover heritable disparity at independent or several gene loci of individual plants within a population or between the plant populations. In modern times, due to attainability of a huge number of disclosed expressed sequence tags (ESTs) several SSRs have been evolved and are mentioned as EST-SSRs^11^. In our study, the total numbers of 73 primers were utilized in dissecting molecular signatures of 9 chickpea genotypes, out of which 12 primers with 0.45 average PIC value showed polymorphism (Supplementary Table 2).

### The novel EST-SSR frequency

Since EST sequences are usually partial length cDNAs, it may be impossible to identify sufficient and suitable sequences to delineate fringing priming coat for the harbored SSRs. The use of CAP3 software facilitated the identification of overlapping sequences among ESTs and generation of consensus contiguous sequences for improving the chance to identify sufficient flanking sequences. Out of the assessed ESTs, 18.4% fell into shared and contiguous sequences (1,178), indicating relatively a high level of redundancy within and between the chickpea EST databases. The type and length of an SSR motif is an important factor in determining its usefulness as a marker, since some motifs are more common leading to a larger repeat, the higher the probability that it will be polymorphic^12^. Within the EST-derived 27 SSR markers or constructed contigs (Supplementary Table 3), the most common repeats were di (CT, TA), tri (AGA, TCA, TGG), tetra (CCAC, ANTC), penta (AAANA, TCTCN), and hexa (AATATT) varying in length from 2 to 10 units.

### Putative functional categorization of the new EST-SSR markers

ESTs are currently the most widely sequenced nucleotides derived from plant genomes in terms of numbers of sequences and available nucleotide counts. Following functional characterization, the identified novel SSR loci may be useful for mapping and possible co-localization with QTLs for desirable traits and for future validation as possible candidate genes^13^. In particular, there is an urgent need to uncover sequences that are physically and functionally associated with traits of interest^14^. Following comparison with sequences within the databases, such as those from the existing EST library^15^, functional annotation of the identified EST-SSR showed homology with proteins associated with various biological processes, molecular functions and cellular components. The designed SSR flanking primer pairs gave amplified products in the expected size range in each of the assessed chickpea genotype and 6 of these produced polymorphisms with a median PIC (0.46) value for the 9 genotypes (Supplementary Table 2). The identified novel SSR loci may be useful for mapping and possible co-localization with QTLs for desirable traits and for future validation as possible candidate genes^13^. In particular, there is an urgent need to uncover sequences that are physically and functionally associated with traits of interest^14^. Following comparison with sequences within the databases, such as those from the existing EST library^15^, functional annotation of the identified EST-SSR showed homology with proteins associated with various biological processes, molecular functions and cellular components. Out of the 27 newly designed SSR markers optimized for amplification, four showed gene ontology for proteins involved in drought stress, one for protein folding and one for molecular function. Further, polymorphic markers sequences encoding ribosomal protein (SVP 146) and dehydrin (SVP 213) genes were also identified (Table 3).

**Table 3:** Functional inference of the EST-derived SSR markers.

S.N	Marker	Ontology of the originating EST	Inferred function	Accession No
1	SVP 2	Specific ABA-and stress-inducible gene (PF03134)	Drought stressed related protein	EH059155
2	SVP 134	The BTB domain (Broad-Complex, Tramtrack and Bric a brac)	Protein folding	FE669933.1
3	SVP146	Ribosomal protein L27 signature	Molecular function	FE669871.1
4	SVP 204	Ribosomal protein S14 signature	Drought stressed related Protein	FE670498.1
5	SVP213	Dehydrin Rab18	Drought stressed related protein	FE672454.1
6	SVP 285	Dehydrin signature	Drought stressed related protein	FE673280.1

The meticulous perusal and interpretations of approximate macro and micro nutrient estimations (Table 1), primers amplification scoring sheet, number of alleles, repeat motifs, product size, polymorphism level and PIC values (Supplementary Table 2 and 3), similarity and dissimilarity matrices (Table 2) identified the potential genotypes as PUSA-1103 for higher carbohydrate and zinc, K-850 for higher antiradical activity and fibre, PUSA-1108 for protein and PUSA-1053 for higher Iron, Zinc along with their respective polymorphic markers namely ICCeM012 for PUSA-1103, PUSA-1053; ICCeM0049 for K-850, PUSA-1108, PUSA-1053; ICCeM0067 for K-850, PUSA-1103, PUSA-1053; ICCeM0070 for K-850, PUSA-1108, PUSA-1103; ICCeM0078 for K-850, PUSA-1108; SVP55 for PUSA-1108, PUSA-1103, PUSA-1053; SVP95 for PUSA-1108, SVP96 for PUSA-1108, PUSA-1103, PUSA-1053; SVP146 for K-850, PUSA-1108, PUSA-1103, PUSA-1053 and SVP217 for K-850, PUSA-1108, PUSA-1103, PUSA-1053. Thus, it is evident that 5 newly designed markers SVP55, SVP95, SVP96, SVP146 and SVP217 are able to express their association with macro–micro nutrients and can be used in selecting parents and breeding lines to improving nutritional traits. The expression of genes associated to the absorption and use of Fe and Zn in chickpea plants are controlled by interrelationship and interactions amongst the all available micronutrients. This process is necessary for the development and expansion of chickpea plants as well as for the production of highquality protein for human consumption. Despite having varied degrees of relatedness, the accessions do not cluster according to their biological components^16^. Hence, we investigated the micronutrients nutritional profiles and their association with EST-SSR markes through modulating the plymorphism analysis, minor allele frequency and heterozygosity level. Thus, a fewmarkers were discovered to have a considerable association with nutritionalor antinutritional features of interest. The markers ICCeM0012, ICCeM0049, ICCeM0067, ICCeM0070, ICCeM0078, SVP55, SVP95, SVP96, SVP146 and SVP217 showed association with nutritional traits including high antiradical active elements, high phytic acid content and fibre content in chickpea. Of the 27 SSR markers optimized for amplification, four showed gene ontology for proteins involved in drought stress, one for protein folding and one for molecular function. Sequences encoding ABA specific (SVP2) BTB domain (SVP 134), ribosomal protein (SVP 146, SVP 204) and dehydrin (SVP 213, SVP 285) genes were also identified (Table 3).

### Number of alleles and molecular polymorphism

**Figure 2:**
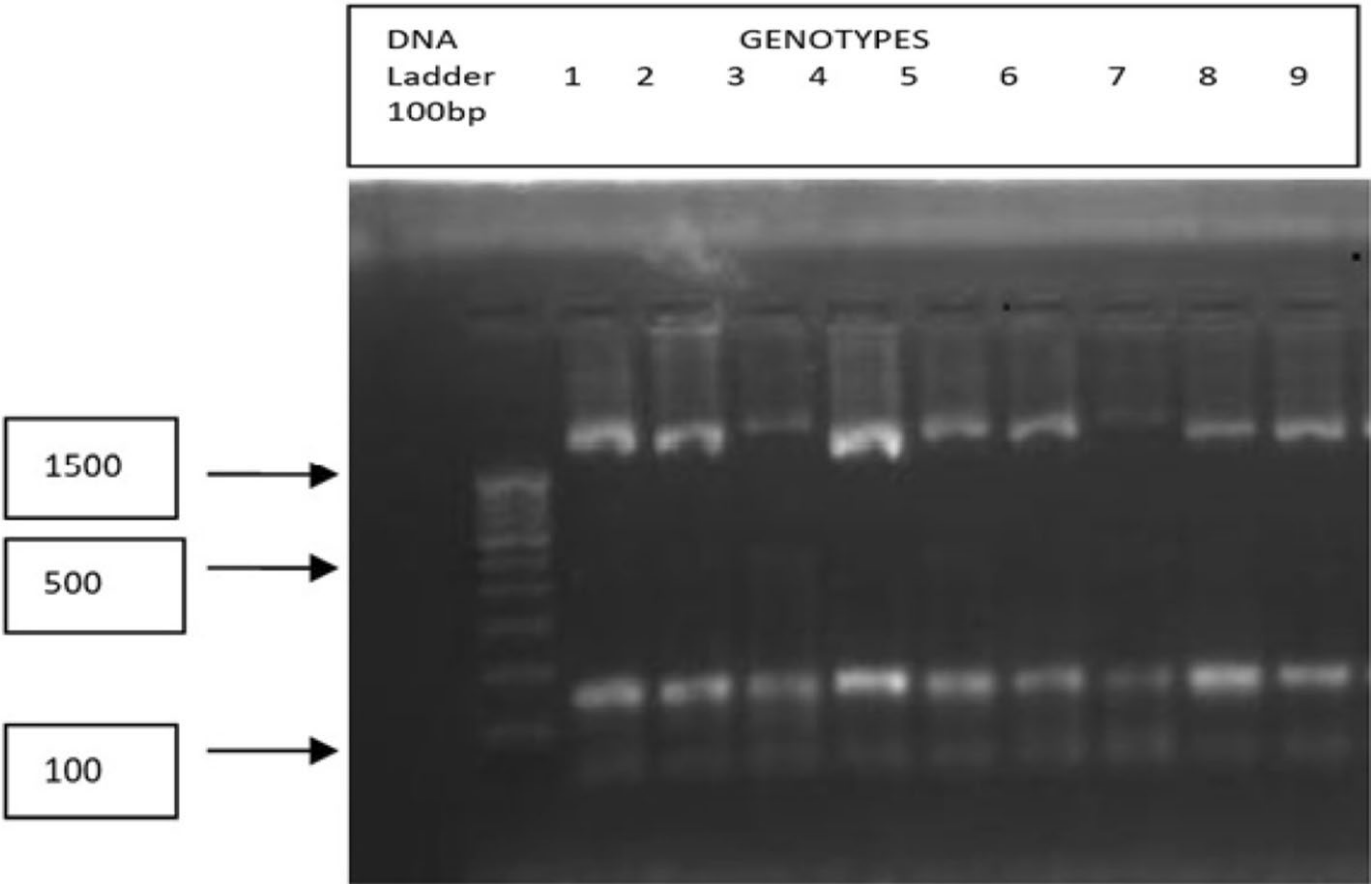
Amplifications expressed by the polymorphic primer ICCeM0059 across the 9 chickpea varieties namely PUSA-362 (1), K-850 (2), PUSA-1105 (3), PUSA-1108 (4), PUSA-1103 (5), JG-62 (6), PUSA-1053 (7), JG-74 (8) and PUSA-1088 (9).

The highest numbers of three alleles were observed for the primers SVP 95 and ICCeM0059 (Fig. 2) followed by SVP 55, SVP 96, SVP146, ICCeM0067, SVP213, SVP217, ICCeM0012, ICCeM0023, ICCeM0049, ICCeM0067, ICCeM0070, ICCeM0078 (Two alleles) and only one allele for other primers. Primer ICCeM0059 had maximum number of sharing alleles i.e., 27 and the primer ICCeM0025 had minimum number of sharing alleles i.e., 4 among the primers showing polymorphism. On the basis of sharing alleles, the frequency of primer ICCeM0059 per allele becomes 0.33, 0.33 and 0.33, while that of ICCeM0025 is 1.0. Based on the allele frequencies, the PIC values were estimated for different EST-SSR primers. The PIC values for the 13 polymorphic markers ICCeM0012, ICCeM0049, ICCeM0059, ICCeM0067, ICCeM0070, ICCeM0078, SVP55, SVP95, SVP96, SVP146, ICCeM0067, SVP213 and SVP217 ranged from 0.28 to 0.68. Amongst the polymorphic markers SVP 213 showed the lowest PIC value (0.28) and ICCeM0059 showed the maximum PIC value (0.68) because of evenly distribution of three alleles among the genotypes of *C. arietinum* (Supplementary Table 1).

### Molecular similarity versus dissimilarity analysis

EST-SSR data were employed to compare pair wise genotypes based on combined and unmatched products with NTSYS-PC-version 2.11 s (Table 2). The aptness of SSRs in discovering intraspecific disparities in chickpea has been illustrated applying polymorphic SSR markers to investigate intra-specific genetic variations amongst geographically distant *Cicer* genotypes^17^. Genomic closeness amongst genotypes was assessed by a similitude grid based on Jaccard’s coefficients ranging 0.76 to 1.00.

**Figure 3:**
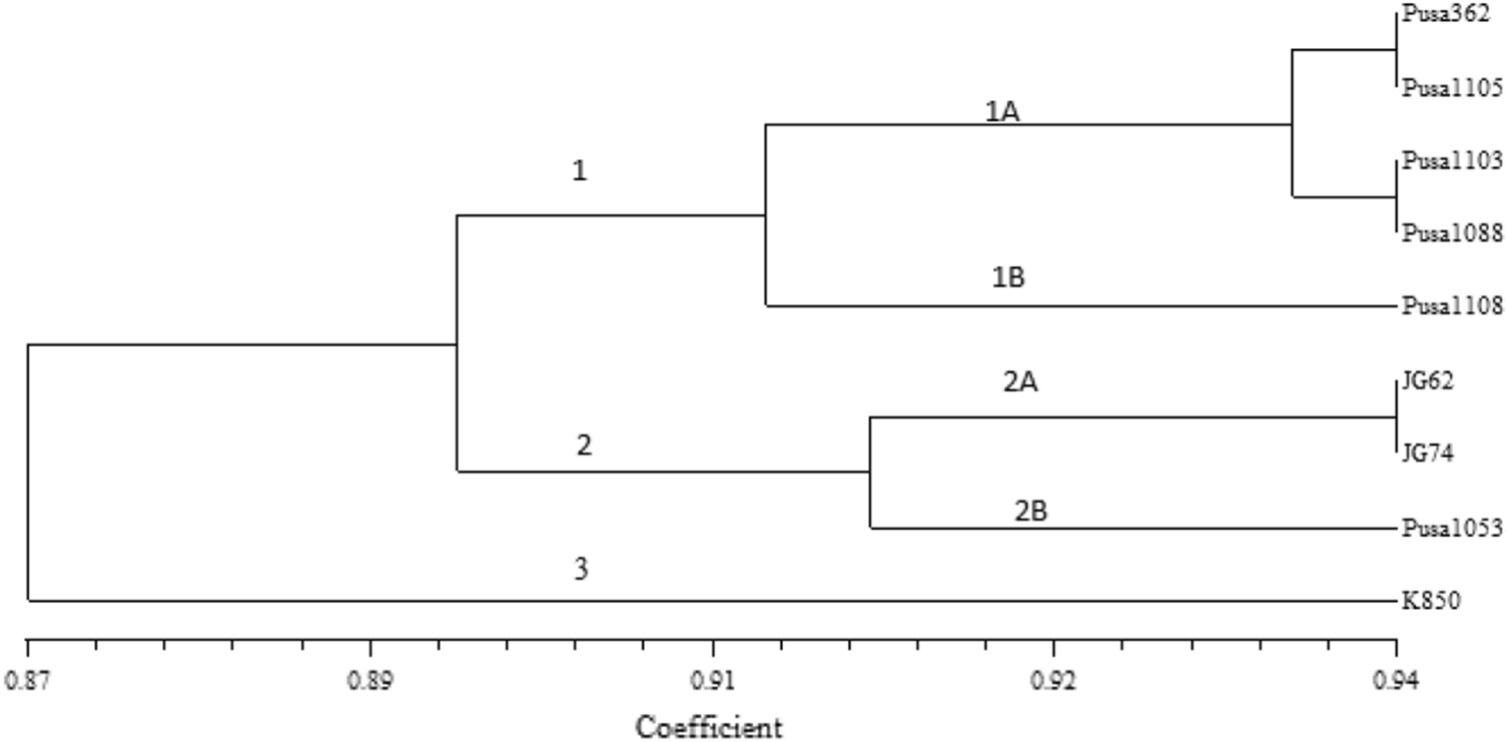
Molecular dendrogram of 9 chickpea varieties constructed by UPGMA cluster analysis based on genetic similarity SSR data.

Molecular profile-based similarity coefficient was applied to cluster the data as per UPGMA algorithm that produced 3 clusters (Fig. 3). The Cluster-1 comprised 5 genotypes that were further aligned into two sub clusters viz. 1A and 1B. Cluster 1A is represented by 4 genotypes revealing the identical genomic similarity coefficient value (0.94) between PUSA-362 versus PUSA 1105, PUSA-362 versus PUSA 1103, Pusa-1088 versus PUSA 1103 and PUSA 1088 versus PUSA 1105. Cluster 1B comprised single genotype PUSA 1108 very closely correlated with cluster 1A. The Cluster-2 comprised 3 genotypes and those were further grouped into two sub clusters viz. 2A and 2B. Cluster 2A comprised genotypes JG-62 and JG-74 having 0.94 genomic similarity coefficient values and cluster 2B contained a single genotype PUSA 1053 expressing close association with cluster 2A. The genotype K-850 remained isolated at the end of the dendrogram.

## Discussion

Developmental and degenerative disorders are known to be primarily caused by damage to the genome. Many micronutrients serve as cofactors or substrates for enzymes that repair, methylate, and synthesize DNA as well as enzymes that detoxify genotoxins, assisting in the prevention of DNA damage events brought on by both endogenous and exogenous sources. Furthermore, it is evident that genotype-dependent nutrient-nutrient and nutrient-gene interactions may have an impact on how either micronutrient excess or deficiency affect genomic stability^18^. Chickpeas exhibit nutritional benefits and are recommended for sustainable diets. Proximate analysis of selected chickpea genotypes revealed that these genotypes possess high macro and micro nutrient contents and show great phytochemical potential. Findings of proximate compositions are in agreement with the studies conducted earlier on legumes by other researchers^1,19–24^. As far as total polyphenols and antioxidant activity are considered, our results showed significantly high level of TPC and antiradical activity which suggests that these genotypes are having substantial phytochemical properties which can be utilized in product development to cure the inflammation and malnutrition. Our results of TPC are in consistent with earlier reports^23,25^ and also showed similarity with chickpea and certain underused legumes in Korea like pigeon pea (248–300 mg), groundnut (140–358 mg), kidney bean (250–320 mg) and groundnut species (100–289 mg) as reported^26^. As co-factors for many proteins, iron (Fe) and zinc (Zn) are essential micronutrients that play important roles in maintaining life^27^. The findings showed that the examined genotype’s seed Fe and Zn content varied significantly. Table 1 lists the range and population median for 12 phenotypic traits in 9 genotypes of chickpea. With the exception of protein, Fe, Zn and tanin, antiradical activity most features have a symmetric distribution of nutrients. Pusa 1108 had high protein (31/100 g) but also contains high phytate, high tannin and high TPC. Pusa 1053 was found to be the best in terms of micro and macro-nutrient content as it has high Protein (28/100 g), high Zn (6.2 mg/100gm), High Fe (10.5 mg/100gm) and low antinutritional factors viz., Low TPC (101 mg/100gm), Phytate (598 mg/100gm), tannin (0.07%) and antiradical activity (67). Hence Pusa 1053 could be best chickpea variety for the human consumption and of breeding value for nutritional traits. In general, all desi had greater antioxidant activity in comparison to kabuli chickpeas. Such discrepancies in antioxidant actions amongst genotypes were additionally found in several studies that can emerge due to genetic variations, the extraction method and external ambient like rainfall, temperature etc.^28^

Our results on frequency and Characterization of Novel EST-SSR showed that the most frequent repeat type was trinucleotide (35.29%) followed by tetra (23.5%) and di nucleotide (18%) motifs. The ample of trinucleotide motifs in the chickpea coding sequences (35.29%) was in concurrence of inspections noted in mono and dicots^29^ emulating the necessity of the coding domains to perpetuate the codons^30^. In total, 348 of the 1,778 contigs encompassed SSRs (19.6%) of which 27 contained ample fringing sequences to blue print primer pairs (Table 1). In a similar study, relatively higher level of EST-SSR (11.5%) from the assessed ESTs in *Cicer arietinum*^31^) as compared to SSRs (3.2%) in cereal^32^ was observed. However, it should be kept in mind that the myriad of SSRs excavated out of a sequence database turns on the SSR discovery criteria, the size of the dataset and the database mining tools that are operated^33^. The 27 SSR flanking primer pairs designed in the current study amplified products in the expected size range in each of the assessed chickpea genotype and 6 of these produced polymorphisms with a median PIC (0.46) value for the 9 genotypes (Supplementary Table 2).

Regarding putative functional categorization of the novel EST-SSRs, the joint mapping and expression studies will determine the potential usefulness of markers for traits of interest. Future approaches will integrate transcriptomics and marker development in a single step. Although the level of polymorphism within EST derived SSR markers is generally lower than within SSR derived from genomic libraries^34^, the markers in our study have shown to be polymorphic across several accessions. The use of SSCP analysis may further disclose internal single nucleotide polymorphisms^35^. In future, the SSRs developed from ESTs will be mapped to determine if they co-segregate with the genetic variation explained by the trait loci as an initial step towards identifying potential candidate genes.

The high PIC value observed by us is also supported^36^. Meticulous perusal and interpretations based on primers amplification, number of alleles, repeat motifs, product size, polymorphism level and PIC values indicated that ten primers namely ICCeM012, ICCeM0049, ICCeM0070, ICCeM0078, SVP55, SVP95, SVP96, SVP146, ICCeM0067, and SVP217 revealed their efficiency as potential markers for macro–micro nutritional trait association and polymorphism studies.

The maximum genomic similarities (0.94) among 9 genotypes were expressed by the genotypes PUSA 362 versus PUSA 1105, PUSA 1103 versus PUSA 362, PUSA1105 versus PUSA 1088, PUSA 1088 versus PUSA 1103 and JG-74 versus JG-62. However, the minimum genomic similarity (0.82) was observed between the genotypes PUSA 1053 and PUSA 1108. The similarity coefficients were employed to congregate the data following the UPGMA algorithm. The consequent depicted phenogram assembled 9 genotypes into 3 distinct clusters with different sub clusters (Fig. 1). Similar works have also been reported^37–41^ utilizing different molecular markers in chickpea.

We applied an integrated approach of macro–micro nutrients and molecular diversity analysis across nine chickpea genotypes. The nutritional analysis revealed maximum variation between PUSA-362 versus PUSA-1053 for the ash, PUSA-1108 versus K-850 for moisture, PUSA-1108 versus PUSA-1103 for protein, PUSA-1105 versus PUSA-1103 for fat, PUSA-1103 versus PUSA-1088 for carbohydrate, K-850 versus PUSA-1108 for fibre, K-850 versus PUSA-1053 for TPC and antiradical activity, JG-74 versus PUSA-1053 for phytate, JG-62 versus PUSA-1053 for tannin, PUSA-1053 versus JG-74 for iron and PUSA-1053 versus PUSA-1088 for zinc contents indicating richness of PUSA-362 for the trait ash, PUSA-1108 for moisture and protein, K-850 for fibre, TPC and antiradical activities, JG-74 for phytate, PUSA-1053 for iron and zinc, PUSA-1103 for carbohydrate. On the other hand, lowest contents were reflected by the PUSA-1103 for the traits; protein and fat, PUSA-1053 for ash, PUSA-1088 for carbohydrate and zinc, PUSA 1053 for TPC, phytate and antiradical activities. The findings are in conformities of the earlier nutritional availability potential for the above varieties K-850, PUSA-1108 and PUSA-1053 as reported^42,43^.

The nutritional dendrogram expressed close association between the desi genotypes (PUSA-362, PUSA-1103 & K-850) by allocating them in cluster-1 and kabuli genotypes PUSA-1053 and PUSA-1108 were allocated in clusters-3 & 4 respectively indicating significant nutritional variation in desi versus kabuli genotypes. However, the molecular dendrogram expressed distant associations amongst the desi (PUSA-362, PUSA-1103 & K-850) and kabuli (PUSA-1053 & PUSA-1108) genotypes by allocating them in clusters 1A, 1A, 3, 2B & 1B respectively and placing PUSA-362 & K-850 at the two ends. Thus, Cluster analysis based on nutritional and molecular diversities partially match to each other in principle and needs an in-depth analysis to extract the advisory conclusion.

The close perusal of nutritional observations revealed overall superiority of PUSA-1103 and K-850 over PUSA-362 in the tune of earlier studies conducted^19^. The genotype PUSA-1103 has also been reported to be a resource donor for nickel and drought resistance^44^.

Thus, an intense scientific interpretation suggested that the identified novel potential resources as chickpea genotypes PUSA-1103 for higher carbohydrate and zinc, K-850 for higher antiradical activity and fibre, PUSA-1108 for protein and PUSA-1053 for higher Iron, Zinc and lower TPC and phytate contents and the 10 EST-SSR markers ICCeM012, ICCeM0049, ICCeM0070, ICCeM0078, SVP55, SVP95, SVP96, SVP146, ICCeM0067, & SVP217 may be utilized as potential donor/marker resources for the macro–micro nutritional trait specific development of mapping populations, construction of genetic maps, marker trait associations, localization of genes/QTLs for the useful nutritional traits in chickpea. Further, the identified genotypes being agronomically adopted varieties may also be utilized by food technologist and govt sponsored product-oriented schemes for amelioration of malnutrition amongst infants, children and pregnant women.

Genomic assisted breeding, which primarily involves the identification and introgression of useful genes/QTLs (quantitative trait loci) regulating various micro and macro-nutrients like, protein, Fe, Zn and vitamin contents, can be used as a potent intervention tool for biofortification of food crops with the development of cutting-edge technologies such as next generation sequencing (NGS)- and array-based genotyping strategies. To raise the bioavailable micronutrients and hence improve the quality component qualities in food crops for nutritional security, the crucial contributions from the aforementioned research would be helpful. A tremendous amount of work is going into understanding the intricate genetic makeup of this feature in many crop species, such as cereals and legumes. In the study conducted at ICRISAT, a wide range of variation in the 12 nutritional qualities was seen in the chickpea reference set. Moreover, SNPs related with the 12 nutritional attributes and prospective donors in chickpea were revealed. With the ultimate goal of genetically enhancing chickpea, the current work has attempted to discover the candidate gene/marker to delineate functionally relevant molecular tags (markers, genes, QTLs, and alleles) controlling seed protein, phytate, tannin, Fe, and Zn concentrations. For the development of nutrient-rich crops, it is essential to comprehend the genetic basis of interactions between micronutrients, such as the synergistic effect of Fe, crude protein, and the vitamin complex and the competitive effect of -carotene and phytic acid with the vitamin complex and bioconversion factors^16^.

## Methods

### Experimental plots

The experimental research and field studies on chickpea were carried out in a randomized block design, as it has edge over several of other designs due to its capacity to eliminate the inequality of soil fertility gradient, observing the national and legislative guidelines in the experimental field (MB 6 B) of Division of Genetics, IARI, New Delhi. The experimental plot was topographically uniform situated at an altitude of 225 m above mean sea level between 28° 38′ 0″ N to 28° 38′ 30″ N latitude and 77° 9′ 0″ E to 77° 9′ 15″ E longitude. The field soil was sandy loam with mild alkaline about 7.5–8.5pH with low EC about 0.4–0.6 dS/m, low organic content (< 0.5%), low nitrogen (< 280 kg/ha), high phosphorous (24–50 kg/ha) and high potassium (> 280 kg/ha), medium sulphur (10–20 mg/kg), adequate zinc (1–5 mg/kg), adequate iron (5.8–10 mg/kg), adequate manganese (10–25 mg/kg) and adequate copper (0.5–10 mg/kg) respectively.

During crop season temperature ranged from 2.4 to 42.0 °C with mean value of 23.0 °C. Rainfall remained between 0.0 and 0.5 mm with an average of 0.1 mm. The wind speed remained 0.2–13.3 kmph with an average of 3.5 kmph. The mean relative humidity varied from 50.0 to 98.0% with an average of 80.6%. Evaporation varied from 1.3 to 8.6 mm with an average of 4.1 mm.

### Plant materials

Present study includes nine agronomically adopted and superior genotypes of *Cicer arietinum* comprising of five dark brown desi (PUSA-1103, PUSA-362, JG-62, K-850, and JG74) and four white Kabuli (PUSA-1105, PUSA-1108, PUSA-1053 and PUSA-1088) genotypes selected for macro, micro nutrients and genomic descriptions having biotic and abiotic for tolerance representing different eco-geographic locations (Supplementary Table 1). Conditions for choosing the genotypes of distant regions were based on the high nutritional contents, wide acceptability, adoptability and availability of enough grains for fortification with deficient micronutrients by food technologist and govt sponsored product-oriented schemes for amelioration of malnutrition amongst infants, children and pregnant women, the already generated ‘passport’ data as well as field examinations recorded over a decade period in the experimental fields of IARI, New Delhi. Healthy seeds of each genotype were cultivated in a ‘randomized block design’ with a set of three repetitions under all suitable agronomic practices during 2020–21. The leaves were used for molecular and seeds for nutritional studies.

### Macro and micro nutrients’ estimation analysis

Protein content of seed samples were evaluated by using Kjeldahl method. 0.5 g of sample was taken and placed into a Kjeldahl digestion flask for the digestion and percent nitrogen was calculated as per AOAC^42^.

Following equation was used to calculate nitrogen percent:

$${\text{Nitrogen}}\;\% = \left( {{\text{Sample}}\;{\text{titre}} - {\text{Blank}}\;{\text{titre}} \times {\text{N}}\;{\text{of}}\;{\text{HCL}} \times {14} \times {1}00/{\text{Weight}}\;{\text{of}}\;{\text{sample}} \times {1}000} \right) \times {1}00$$

Thereafter, protein content was measured by the equation: Protein% = 6.25 × Nitrogen%

Fat content was determined by Soxhlet method by dissolving 2 g of seed sample in petroleum ether as per AOAC^42^.

Fat content in percentage was calculated after complete extraction of the sample by using following equation:

$${\text{Fat}}\% = \left( {{\text{Weight}}\;{\text{of}}\;{\text{beaker}}\;{\text{with}}\;{\text{oil}} - {\text{Weight}}\;{\text{of}}\;{\text{pre - weighed}}\;{\text{constant}}\;\left( {{\text{blank}}} \right)\;{\text{beaker}}/{\text{Weight}}\;{\text{of}}\;{\text{sample}}} \right) \times {1}00$$

Carbohydrate determination was done by difference method^45^ and calculated by the following formula:

$${\text{Carbohydrate}}\;(\% ) = 100 - \{ {\text{weight}}\;{\text{in}}\;{\text{grams}}\;({\text{protein}} + {\text{fat}} + {\text{moisture}} + {\text{ash}} + {\text{crude}}\;{\text{fibre}})\;{\text{in}}\;100\;{\text{g}}\;{\text{of}}\;{\text{the}}\;{\text{food}}\;{\text{sample}}$$

Crude fibre was extracted with petroleum ether (2 g of samples were used) and residual fat free sample was used for fiber estimation as per AOAC^46^.

The percent loss in weight was expressed as crude fibre.

$${\text{Crude}}\;{\text{Fibre}}\;\left( \% \right) = \left\{ {\left( {{\text{W1}} - {\text{W2}}} \right)/{\text{W}}} \right\} \times {1}00$$

where W = Weight of sample (g), W_1_ = Crucible weight after oven drying (g), W_2_ = Crucible weight after ashing (g).

Determination of Total polyphenol content (TPC) was done by Folin-Ciocalteu method as described in ISO 14502-1:2005 (E)^47^.

Phytate content of the selected sample was determined by the ferric nitrate method^48^. By using Ferric Nitrate, a standard graph was plotted to calculate micrograms of iron by following expression:

$${\text{Phytate}}\;\left( {{\text{mg}}/{1}00\;{\text{g}}} \right)\;{\text{of}}\;{\text{sample}} = \left\{ {\mu \;{\text{Fe}}/{\text{Weight}}\;{\text{of}}\;{\text{sample}}\;\left( {\text{g}} \right)} \right\} \times {15}$$

The antiradical activities of sample extracts were assessed by DPPH (2, 2-diphenyl-1-picrylhydrazyl radical) method^49^ with slight modification. The percent anti radical activity was calculated using following formula:

$${\text{Antiradical}}\;{\text{activity}}\;\left( \% \right) = \left\{ {\left( {{\text{Control}}\;{\text{Absorbance}} - {\text{Sample}}\;{\text{Absorbance}}} \right)/{\text{Control}}\;{\text{Absorbance}}} \right\} \times {1}00$$

Tannin content was estimated by Folin-Denis method^48^ by using tannic acid as a standard. A curve was plotted to measure tannin (%) as tannic acid.

Iron and zinc concentration in samples were determined according to standard procedure^46^ by using atomic absorption spectrophotometer (AAS). Standard curves of iron and zinc (NIST) were standardized and concentrations of minerals were determined as mg/100 g.

Ash content was estimated from 2 g seed samples on dry weight basis for each variety as per procedure^46^.

Moisture content was assessed from 3 g seed samples for each genotype as per the procedure described^46^.

### Statistical data analysis for macro and micro nutrients

SPSS version 7.5 software was used for all analysis of the nutritional evaluations stated as means of three repetitions. The outcomes were scrutinized by one way analysis of variance (ANOVA), followed by Duncan’s multiple range tests to compare means significance at *p* < 0.05.

### Designing of new EST-SSR markers

The availability of genomic resources like molecular markers and linkage maps shows how considerably chickpea genomics research has advanced recently with the emergence of new techniques^50^.The creation of functional genic molecular markers (GMMs), which are obtained from transcript sequences, has become increasingly important with the recent increase in emphasis on functional genomics research in a number of species. In this context, the growing EST databases have offered a desirable resource for the creation of several types of efficient EST-based markers, including EST-SSRs, intron targeted primers (ITPs), and expressed sequence tag polymorphisms (ESTPs). Single nucleotide polymorphisms (SNPs), which are thought to be the most common type of genetic variation, have lately been proposed as being particularly well suited for a variety of applications, including increased marker density, QTL mapping, and high-throughput marker-assisted selection. These several classes of EST-based markers all come from genes with established functions and offer a way to map the genome's gene-rich areas. The goal of this work was to create markers linked to the dietary characteristic and increase the supply of EST-based genic markers. There are almost no reported markers for nutritional traits in chickpea. Further, designing genomic SSR markers are a huge and herculean task. Hence, the chickpea EST sequences available in the NCBI database^51^ were downloaded in FASTA format (Accession No. CDO 38847-GR 394575). These EST sequences were clustered into contigs and singletons using CAP 3 software. The resultant 348 contigs were mined for novel SSRs using tandem repeat occurrence locator (TROLL) analysis to explore for dinucleotide, trinucleotide, tetranucleotide, pentanucleotide and hexanucleotide repeat motifs. The EST-SSR markers/primer pairs were designed using Primer 3 software^52,53^ by following the optimal parameters: 40–80% GC content, 50–60 °C Tm; 15–25 bases primer length and 100–280 bp product length. The primers were synthesized by Imperial Life Sciences, USA and designated as SVP with a numerical identification.

### DNA isolation, amplification and detection of microsatellite alleles

Isolation of Genomic DNA was done from young leaves as per CTAB method^54^ with few modifications. 3 gm of sample (fresh leaves) was taken to make fine powder with the help of mortar, pestle and liquid nitrogen. The fine powder was then shifted to a centrifuge tube carrying 15 ml of warmed (65 °C) extraction buffer. Occasional shaking was done to mix the samples thoroughly. Thereafter, samples were incubated for 60 min at 65 °C. An equal volume of chloroform: isoamyl alcohol (24:1) was added to each tube and mixed gently for 15–20 min. When the mixing was done, tubes were centrifuged for 10 min at 8000 rpm (CPR-24, Remi India). Aqueous phases were shifted to blank tubes and again extracted with chloroform: isoamyl alcohol. After that, chilled isopropanol (0.6 ml) was mixed and tubes were kept at − 20 °C for 2 h. Again, the tubes were centrifuged for 15 min at 4° C at 10,000 rpm. After centrifugation, supernatants were rejected and pellets were cleaned using 70% ethanol. Finally, the pellets were air dried and dissolved in 100 µl of TE buffer.

Eppendorf Master Cycler gradient was used for carrying out PCR analyses, in a total volume of 10 µl containing10 × buffers of 2.5 mM MgCl_2_, 25 ng of template DNA, 10 mM dNTPs, 10 µM of primers, 0.5 U/µl Taq DNA polymerase enzymes (BangloreGenei). 73 EST-SSR markers comprising of newly designed 27 EST-SSR markers by us, 39 & 7 markers designed^12,55^ were used for amplification. Further for performing PCR analysis after initial denaturation for 2 min at 95 °C, 35 cycles of cycling protocol consisting of denaturation at 95° C for 20 Sec, annealing at 52–70 °C for 50 Sec and elongation at 72 °C for 50 Sec were used. Final extension of complete cycle was done at 72° C for 7 min. 1.2% agarose gel carrying ethidium bromide (0.5 µg/ml) in 1 × TBE buffer was run at 60 V to resolve amplicons. The amplified products were double rechecked for their reproducibility for each polymorphic primer. Calculations of frequencies of incidence of all polymorphic alleles were done for the determination of polymorphic information content.

A CCD camera assembled to a gel documentation system having the quantity one software (Alpha Innotech) was used to take photograph of the gel. Scorings were accomplished manually for each of the gel sections and alleles were determined on the basis of the positions of bands. Band pattern for each of the microsatellite markers was documented for each genotype by assigning a letter to each band. All the alleles were numbered as ‘*a1*’, ‘*a2*’ etc. In the data matrix, occurrence of a band was denoted as ‘1’ and absentia of a band was denoted as ‘0’. The efficacies of 73 markers were measured by polymorphic information content (PIC) as per assessment procedure^56^.

$${\text{PIC}} = {1} - \sum {\text{p}}_{{{\text{ij}}}}^{2}$$

where P_ij_ is the frequency of the *j*th allele for *i*th locus summed across all alleles in the locus.

The pairwise genomic resemblances for all the genotypes were assessed as per Jaccard’s coefficient^57^ and all statistical analysis were accomplished utilizing the software NTSYS-PC (version 2.11 s)^58^.

### Functional annotation of the new EST-SSR markers

Functional annotation of newly designed markers was obtained from GenBank using the blast X algorithm against the nr database^59^. The contigs employed for marker development were deciphered using TranSeq^60^. The derived putative amino acid arrangements were given into for a domain search in gene ontology^60^ and GO Terms were withdrawn from the top most identical hits^61^. The AmiGO term browser^56^ (http://amigo.geneontology.org/cgibin/amigo/search.cgi) was used to find molecular function, cellular compartmentalization and inferred biological process ontology. The Pfam database was used to infer gene function^62^.

### Clusters analysis for measurement of distances

Software NTSYS-PC version 2.11 s was employed to categorize genotypes into discrete conglomerations^23^ followed by dendrograms constructions using the UPGMA method^63^.

## Supplementary Information

Supplementary Tables.

## Data Availability

All data generated or analyzed during this study are included in this published article (and its Supplementary Information files).
