# Supplementary material for: Disentangling potential genotypes for macro and micro nutrients and polymorphic markers in Chickpea
Source: Sci Rep. 2023 Jul 3;13:10731. doi: 10.1038/s41598-023-37602-2 (PMC10318058; doi:10.1038/s41598-023-37602-2)
Supplement: Supplementary file 1 — Supplementary Tables. [file 41598_2023_37602_MOESM1_ESM.pdf]

Supplementary Table-I: Chickpea Varieties used as experimental material

S. No.	Genotype	Major Characteristics	Origin / Source
1.	PUSA- 362	Desi, developed through hybridization (BG-303 x P-179), released in 1995, tall, maturity (145-150 days), wilt resistant, bold seeded, seed yield (23-24 q/ha) and suitable for north-west plain zone.	Indian Agricultural Research Institute, New Delhi, India
2.	K-850	Desi, developed through hybridization (Banda Local x Etah bold), released in 1982, medium tall, erect, maturity (145-150 days), large dark greenish foliage, seeds reddish brown, round, smooth, bold (24g / 100 seeds), seed yield (25-28 q/ha) and suitable for northern plain zone.	C.S.A. University of Agriculture and Technology, Kanpur, Uttar Pradesh, India
3.	PUSA 1105	Kabuli, released in 2004, developed through hybridization / pedigree method {(C-104 x BG-1003) x (ICC-88503xBG-1048}, maturity (145 days in north & 118 days in central), yield (25-30 q/ ha) and suitable for Delhi region.	Indian Agricultural Research Institute, New Delhi, India
4.	PUSA 1108	Kabuli, released in 2005, developed through hybridization / selected bulk method {(BG-315 x ILC) x (ICCV-13 x Flip-85-11} x (ICCV-32 x Surutoto-77), bold seeded (29g / 100 seeds), moderate resistant to root diseases, tolerant to pod borer, maturity (145-150 days), yield (29 q/ ha) and suitable for Delhi region.	Indian Agricultural Research Institute, New Delhi, India
5.	PUSA-1103	Desi, released in 2004, developed through hybridization from pedigree method {F1 (Pusa-256 x *Cicer reticulatum*) x Pusa-362}, maturity (117-135days), moderately resistant to soil borne diseases, tolerant to pod borer and bruchid, yield (19-23 q /ha) and suitable for Delhi region.	Indian Agricultural Research Institute, New Delhi, India
6.	JG-62	Desi, released in 1972, seeds medium, yellowish brown, double seeded, early maturity (about 120 days), yield (16-20 q/ ha) and best for short dry growing seasons in Karnataka, Maharashtra, Orissa and western parts of Madhya Pradesh.	J.N.K.V.V., Jabalpur, India
7.	PUSA 1053	Kabuli, developed through hybridization (ICCV-3x FLIPP 88-20), released in 1999, tall, semi erect, white seeds, bold seed size, test weight (27 gm / 100 seeds), seed yield (17-19 q/ ha), wilt tolerant and suitable for north west plain zone.	Indian Agricultural Research Institute, New Delhi, India
8.	JG-74	Desi, composite from genetic stock lines released in	J.N.K.V.V., Jabalpur,

		1976, seed coat puckered, wilt resistant, seed yield (13-15 q/ha) and suitable for Madhya Pradesh.	India
9.	PUSA 1088	Kabuli, released in 2003, medium early, resistant to wilt and root rots diseases, yield (25-30 q/ ha) and suitable for Delhi region.	Indian Agricultural Research Institute, New Delhi, India

Supplementary Table II: Polymorphism Information Content (PIC) of SSR loci across 9 chickpea varieties

S.No.	Primers	Allele Numbers	No. of genotypes sharing alleles	Frequency of SSR alleles	No. of genotypes showing amplification	PIC
1.	ICCeM001	a5	9	1.00	9	0.00
2	ICCeM003	a8	9	1.00	9	0.00
3	ICCeM007	a6	9	1.00	9	0.00
4	ICCeM009	a1	9	1.00	9	0.00
5	ICCeM0012	a1<br>a7	7<br>3	0.78<br>0.21	7	0.34
6	ICCeM0025	a1	4	1.00	4	0.00
7	ICCeM0026	a6	9	1.00	9	0.00
8	ICCeM0030	0	0	0.00	0	0.00
9	ICCeM0031	0	0	0.00	0	0.00
10	ICCeM0032	a5	9	1.00	9	0.00
11	ICCeM0033	a7	9	1.00	9	0.00
12	ICCeM0035	a7	9	1.00	9	0.00
13	ICCeM0036	a5	8	1.00	8	0.00
14	ICCeM0037	0	0	0.00	0	0.00
15	ICCeM0038	a7	9	1.00	9	0.00
16	ICCeM0039	a6	9	1.00	9	0.00

17	ICCeM0040	a5	9	1.00	9	0.00
18	ICCeM0042	0	0	0.00	0	0.00
19	ICCeM0046	a1	9	1.00	9	0.00
20	ICCeM0049	a2<br>a4	6<br>3	0.75<br>0.25	6	0.37
21	ICCeM0050	a6	9	1.00	9	0.00
22	ICCeM0051	a4	9	1.00	9	0.00
23	ICCeM0054	a8	9	1.00	9	0.00
24	ICCeM0056	0	0	0.00	0	0.00
25	ICCeM0059	a6<br>a7<br>a8	9<br>9<br>9	0.33<br>0.33<br>0.33	9	0.676
26	ICCeM0063	0	0	0.00	0	0.00
27	ICCeM0064	a8	9	1.00	9	0.00
28	ICCeM0065	0	0	0.00	0	0.00
29	ICCeM0066	0	0	0.00	0	0.00
30	ICCeM0067	a1<br>a5	6<br>6	0.50<br>0.50	6	0.50
31	ICCeM0070	a5<br>a8	6<br>9	0.33<br>0.67	9	0.45
32	ICCeM0072	0	0	0.00	0	0.00
33	ICCeM0073	a1	9	1.00	9	0.00
34	ICCeM0074	0	0	0.00	0	0.00
35	ICCeM0078	a5<br>a8	4<br>9	0.22<br>0.78	9	0.35
36	ICCeM0089	a5	6	1.00	6	0.00
37	ICCeM0096	0	0	0.00	0	0.00

38	ICCeM0098	a7	9	1.00	9	0.00
39	ICCeM0099	0	0	0.00	0	0.00
40	SVP2	a5	9	1.00	9	0.00
41	SVP3	a5	9	1.00	9	0.00
42	SVP6	a5	9	1.00	9	0.00
43	SVP11	a5	9	1.00	9	0.00
44	SVP16	a5	9	1.00	9	0.00
45	SVP40	a5	9	1.00	9	0.00
46	SVP55	a5<br>a8	9<br>8	0.55<br>0.45	9	0.50
47	SVP59	a7	9	1.00	9	0.00
48	SVP62	a7	9	1.00	9	0.00
49	SVP64	a7	9	1.00	9	0.00
50	SVP66	a7	7	1.00	7	0.00
51	SVP68	0	0	0.00	0	0.00
52	SVP76	a7	8	1.00	8	0.00
53	SVP77	a5	9	1.00	9	0.00
54	SVP82	a8	9	1.00	9	0.00
55	SVP95	a1<br>a2<br>a3	8<br>1<br>9	0.48<br>0.04<br>0.52	9	0.48
56	SVP96	a4<br>a6	7<br>8	0.44<br>0.56	8	0.50
57	SVP118	a5	9	1.00	9	0.00
58	SVP134	a3	9	1.00	9	0.00
59	SVP146	a6<br>a8	9<br>7	0.61<br>0.39	9	0.48

60	SVP147	0	0	0.00	0	0.00
61	SVP162	a5	8	1.00	8	0.00
62	SVP180	0	0	0.00	0	0.00
63	SVP181	a6	9	1.00	9	0.00
64	SVP194	a5	9	1.00	9	0.00
65	SVP204	a5	9	1.00	9	0.00
66	SVP213	A5<br>a8	3<br>9	0.16<br>0.83	9	0.28
67	SVP217	a2<br>a7	9<br>9	0.50<br>0.50	9	0.50
68	SVP219	a5	9	1.00	9	0.00
69	SVP221	a5	9	1.00	9	0.00
70	SVP254	a5	9	1.00	9	0.00
71	SVP285	a5	9	1.00	9	0.00
72	SVP291	a5	9	1.00	9	0.00
73	SVP329	a6	7	1.00	7	0.00

Supplementary Table-III: In Silico designed new EST- SSR molecular markers /primers

S.No.	Primers	Primer Sequence	Repeat Motif	Product range (bp)	Tm Value (°C)
1.	SVP2	F TGAATAAGGTCGTACTGGCT<br>R CTTCCTCTCATAAATGGCAC	(AAGCCA)2	200	56
2.	SVP3	F GTGGGTTGAAGGTATTGAAA<br>R AGCACAGTTGGAGTAAGCAG	(TAG)5(TCA)4	200	56
3.	SVP6	F GCTCGTTTGATTCTGATTTC<br>R TCCCTGTGGTAACTTTTCTG	(CCAC)3	200	55
4..	SVP16	F TCTCAGTTCCCTCATTCAAC<br>R ATTTCTCCCACCAGTCTTTT	(ACC)5<br>(AATTCC)3	200-600	55
5.	SVP40	F GCCGAGGTACTATACGCCAAT<br>R TGGTTCTACAAAAGCTCGTGG	(G)10	100	62
6.	SVP55	F AGAGGCGTTCAGTCATAATC<br>R GAAAGTGGAAGATGAGGTTG	(TCCTC)3	_	56
7.	SVP59	F CTTGCACACAAAAGCAATAG<br>R GCACCATCTTTGACCTTAAC	(TAA)5	400	55
8.	SVP62	F GTCAATCCTTACTATGTCTGG<br>R GGACAAAATCCAATGTGAAC	(TAAAA)3	_	57
9.	SVP66	F GTGATGGAATACTGTGGTGGT<br>RCAAAATCCCCTAGCTTGACATC	(TGAGAT)2	_	60

10.	SVP68	F GCAGGAGTTTTGATTGTATCC<br>R CTGAAGGAGTGGGAGAAGCTA	(TGCTT)2	200	62
11.	SVP76	FAATTTAGAGCCTGAGAGCGTGA<br>R CTGTGAACTTAATCGTGGGGTT	(GATTCAT)2	200	60
12.	SVP77	F TAGCTTGTGGTCTCCATTCT<br>R CTGCATACGAACCTTGATTA	(CCAC) 3	300	55
13.	SVP82	F AACGCAATTCCTATTGAGC<br>R AGGGTTTTGTTAGGGTTTTC	(CT)6	100	53
14.	SVP95	F AGTACATGAGTGAGGCTGCT<br>R CTAGCTGTTGGGTCGAAGT	(ATC)5	_	54
15.	SVP96	F AATTCGTCACGGATGAGAGG<br>R TAATGTCATCGACGGGAACA	(AT)20	150	57
16.	SVP118	F CAATCCTGCACATGAATCT<br>R ACCTCGGCTCTCTCATTTA	(TAAT)3	100	53
17.	SVP134	F TTGCCTAGTTTCTCATTTCC<br>R TCTCTTCTGCTTCCATGATT	(ATTC)3	300	56
18.	SVP146	F GTTTCAACATAGCAACAGCA<br>R CTCCCTTCTTATGAGCATTC	(TCTTCA)3	100-200	54
19.	SVP147	F TGTTTTCACCTTATACGAGC<br>R ATGTAGGTTTGGATGATGG	(AT)6	_	51

20.	SVP194	F CAATAAGAACAGCAGCATCA<br>R ATTTGAGAAGGTTAGGACCC	(CTTT)5	200	55
21.	SVP204	F CACAAGGTGTAATTGCTGAA<br>R AATTTGAGAATGTGGTGAGG	(TAT)4	200	54
22.	SVP213	F GGTATCATCGGTTGTTCCAAA<br>R CACACAGGTAGTGGTGGTGG	(TAT)5	150	53
23.	SVP217	F AGGTTTTGTTGAAGGAGGCA<br>R ATGGAAGTTGTTCCGGTTTG	(CAC)3	200	56
24.	SVP254	F TGTTGGTCCCATAACTACCT<br>R AGCCACATAAACATGGAGAT	(TAT)4<br>(CCA)5	300	58
25.	SVP285	F AGCCACATAAACATGGAGAT<br>R TGTTGGTCCCATAACTACCT	(ATCATA)2	150	56
26.	SVP291	F TTATGGCCTGTATGTCTTCC<br>R AAGAGGCAGGTTGTTTATGA	(GTG) 5 (TA)5	900	55
27.	SVP329	FGGTCGGAAAACGATGTTGAC<br>R TAGGGACAGTGGGAATCTCG	(AT)8	_	59
